# Accelerating wound healing by biomineralizing crystallization formed from ZIF-8/PLA nanofibers with enhanced revascularization and inflammation reduction

**DOI:** 10.3389/fbioe.2025.1629244

**Published:** 2025-09-05

**Authors:** Xiangsheng Wang, Xueer Zhang, Bingqian Wang, Zhixiang Tan, Mulan Chen, Haiyan Shen, Hanxiao Cheng, Zhentao Zhou, Zhanyong Zhu, Jing Guo, Jufang Zhang

**Affiliations:** ^1^ Department of Plastic and Aesthetic Surgery, Affiliated Hangzhou First People’s Hospital, School of Medicine, Westlake University, Hangzhou, Zhejiang, China; ^2^ School of Clinical Medicine, Chengdu University of Traditional Chinese Medicine, Chengdu, China; ^3^ Department of Dermatology, Hospital of Chengdu University of Traditional Chinese Medicine, Chengdu, China; ^4^ Department of Plastic Surgery, Union Hospital, Tongji Medical College, Huazhong University of Science and Technology, Wuhan, China; ^5^ Department of Plastic Surgery, Renmin Hospital of Wuhan University, Wuhan, Hubei, China

**Keywords:** nanofibers, metal-organic framework, polylactic acid, biomimetic mineralization, wound healing

## Abstract

**Introduction:**

Polylactic acid (PLA) is a synthetic polymer material with good biodegradability, biocompatibility, and bioabsorbability, electrospinning is a convenient and efficient method for preparing PLA nanofibers as wound dressing. However, PLA nanofibers as wound dressings lack biological functions, including promoting angiogenesis, extracellular matrix secretion and regulating inflammation, which are crucial for skin regeneration. Herein, we aimed to develop an effectively methods to enhance biological activity of PLA nanofibers through biomimetic mineralized induced by Zeolite imidazolate framework-8 (ZIF-8) for promoting wound healing.

**Methods:**

The ZIF-8/PLA nanofibers were prepared by electrospinning and immersed in simulated body fluids (SBF) to obtain mineralized PLA nanofibers (mZIF-8/PLA). The physicochemical and mechanical properties, Ions releases, and biocompatibility of the mZIF-8/PLA nanofibers were evaluated in vitro. The regeneration capability of the nanofibers was systemically investigated in vivo using the excisional wound-splinting model in Rats.

**Results:**

Hydroxyapatite-like crystals was observed on the surface of nanofibers, EDS-mapping confirmed that the crystal deposits in mZIF-8/PLA nanofibers are composed of calcium, phosphorus, and zinc elements. The mineralized crystallization increased the roughness of PLA nanofibers by altering its surface topography, and significantly improved its mechanical property and hydrophilicity. Biomimetic mineralized mZIF-8/PLA nanofibers significantly improve the biological activity for promoting fibroblast proliferations. The Zinc and calcium ions released from hydroxyapatite-like crystals induced by ZIF-8 also promotes angiogenesis, enhances extracellular matrix deposition and reduces inflammatory infiltration in wound healing model.

**Conclusions:**

In summary, this study demonstrates that mineralized ZIF-8/PLA nanofibers could promote wound healing through regulating angiogenesis and reducing inflammatory response.

## 1 Introduction

Acute and chronic trauma, such as burns and diabetes, remain persisting challenges that cause a huge burden on the patients and healthcare systems worldwide (Xiang et al., 2024). Trauma compromises the integrity of the skin, followed by an intrinsic healing response that protects the injured skin. The wound healing process consists of four broad phases: (i) hemostasis, (ii) inflammation, (iii) proliferation, and (iv) maturation. Hemostasis, which is the first phase of wound healing, begins at the onset of injury and lasts for only a few minutes. The inflammation phase occurs simultaneously with hemostasis, at this stage, neutrophils and phagocytes from blood vessels penetrate into the wound area to kill bacteria and clear debris. The proliferation phase involves epithelial cells and macrophages covering lesion, while fibroblasts and endothelial cells simultaneously move towards the damaged areas, forming granulation tissue composed of new matrix and blood vessels. The maturation phase varies significantly from wound to wound, and can last for several months (Han and Ceilley, 2017; Patel, Srivastava, Singh and Singh, 2019). This complex wound healing process enabled skin to self regenerate, however, this ability is greatly compromised under full-layer injury, and the demand for grafts or dressings is inevitable (Tran, Shahriar, Yan and Xie, 2023).

Recent studies have explored new bioengineering strategies to improve the treatment of patients with skin lesions, moving away from traditional approaches such as autografts or allografts (Yu et al., 2019). An ideal wound dressing should effectively accelerate the healing process, promote rapid vascularized skin formation, prevent scarring, and aid in the reconstruction of multiple tissue layers in full-thickness cutaneous defects. The production of electrospun nanofibers based on biocompatible polymers for skin tissue regeneration has been explored in recent years, due to their capacity to mimic the nanostructure of the natural extracellular matrices (Parham et al., 2020; Wang et al., 2021). Their highly porous structure and spatial interconnectivity are essential for nutrient and waste transport and cell communication, thus supporting cellular phenomena such as adhesion, differentiation, and proliferation, making electrospun nanofibers well-suited as wound dressing material (Jang et al., 2023; Jayarama Reddy et al., 2013; Schröder et al., 2017). The production of electrospun nanofiber materials, ranging from natural to synthetic polymers, has been engineered (Bi et al., 2020; Memic et al., 2019; Tanet al., 2022). Polylactic acid (PLA), which is widely used in the biomedical field and includes degradable sutures, drug delivery materials, nanoparticles, and porous nanofibers, shows great potential for application in the design of wound dressings owing to its excellent biocompatibility, low antigenicity, and controlled biodegradability (Castro-Aguirre et al., 2016; Tajbakhsh and Hajiali, 2017). In this regard, several studies have shown that hybrid PLA-based nanofibrous wound dressings possess interesting features that accelerating wound healing (Echeverría et al., 2019; Fan and Daniels, 2021; Ignatova et al., 2009). However, the PLA dressings suffer from some limitations for their wide application, such as hydrophobicity, insufficient mechanical strength and lack of biological activity (Chen et al., 2023).

Several hybrid formulations have been developed to overcome the shortcoming of PLA biopolymers, particularly the poor biological activity (Hamed et al., 2023; Liao et al., 2023; Mutlu et al., 2023). Zeolite imidazolate framework-8 (ZIF-8) is a subclass of metal-organic framework (MOF) materials that are composed of zinc ions (Zn^2+^) and organic ligands of imidazole derivatives, gain specific interest for wound dressing applications owing to its facile synthesis, high chemical and thermal stability, unique porous structures, accelerated wound healing, and excellent antibacterial properties (Cheng et al., 2024; Xia et al., 2022; Yin et al., 2023). In addition, the released Zn^2+^ can modulate the immune response near the wound site and promote healing (Zhang et al., 2023). For ZIF-8 to be better applied in living organisms, its degradation characteristics should be clarified. As the biological environment is extremely complicated, some aqueous buffered systems have been adopted for *in vitro* and *in vivo* research. ZIF-8 decomposition and collapse can be observed in phosphate-buffered saline (PBS), where phosphates have a high affinity for Lewis metal clusters, which changes the coordination equilibrium to form insoluble zinc phosphates, thereby promoting the release of 2-methylimidazole (2-HmIM) (Bauzá et al., 2016). Moreover, competitive binding may allow anion exchange with ZIF-8 in media rich in metallic cations and inorganic anions (Wang Y. et al., 2020). Compared to PBS, simulated body fluid (SBF) solution contains large amounts of active metal cations and phosphates, as well as attached crystals and charged substrate regions. These regions within SBF serve as nucleation sites that facilitate crystal growth through the conversion of high calcium ion (Ca^2+^), trivalent phosphate ion (PO_4_^3-^), and amorphous calcium phosphate contents into regular carbonated apatite (Salimi, 2021). We previously reported a phenomenon in which ZIF-8 formed large hydroxyapatite-like crystals when immersed directly in SBF. The same phenomenon was observed on the ZIF-8/poly (epsilon-caprolactone) (PCL) composite surface, which improved the *in vivo* and *in vitro* osteoinductivity and biocompatibility of ZIF-8/PCL scaffold (Wang et al., 2023). However, the pro-biomineralization property makes ZIF-8 applicable in polylactic acid-based biomaterials, and its use for skin regeneration is still unknown.

It has been described that calcium ions play an important role in the regeneration of connective tissue and repair of skin, activation of metalloproteinases, as well as keratinocyte growth and differentiation (Bikle, 2023; Bikle et al., 2012; Kawai et al., 2011; Subramaniam et al., 2021). Ribeiro et al. used hydroxyapatite embedded in collagen nanofibers for skin regeneration (Ribeiro et al., 2021), suggested that hydroxyapatite coatings not only have the potential for bone regeneration, but also for skin tissue regeneration. Herein, we speculate that these hydroxyapatite-like crystals induced by ZIF-8 possessed the function of regulate cell function to promote wound healing in the PLA nanofibers.

In this study, ZIF-8/PLA nanofibers were fabricated via electrospinning and soaked in SBF. Large hydroxyapatite-like crystals were observed on the surface of ZIF-8/PLA nanofibers. This biomimetic mineralization induced by ZIF-8 increased the roughness of the PLA nanofibers by altering their surface topography and significantly improving their mechanical and hydrophilicity. *In vitro* experiment showed that mineralized ZIF-8/PLA (mZIF-8/PLA) nanofibers have better biocompatibility and cell proliferation vitality. The mZIF-8/PLA nanofibers significantly promotes angiogenesis, extracellular matrix regeneration and reduces inflammatory infiltration in wound healing model.

## 2 Materials and methods

### 2.1 Preparation of ZIF-8 and ZIF-8/PLA nanofibers

ZIF-8 was synthetized as previously described. Briefly, Zinc nitrate hexahydrate (1.68 g) in 20 mL ethanol solution was slowly added to 4 g of 2-methylimidazole dissolved in 40 mL of ethanol solution under continuous stirring. The reaction solution with a molar composition of Zn2+: 2- HmIM: ethanol = 1:10:30 was stirred at room temperature for 2 h. Then, ZIF-8 powders were isolated by centrifugation at 8500 rpm, washed twice in methanol, and dried at 60 °C for 24 h. TEM samples were prepared by dropping an ethanol dispersion of ZIF-8 onto carbon-coated copper grids; images were acquired on a JEM-2100F (200 kV) and particle size (n = 50) was measured with ImageJ.

PLA and dissolving agent hexafluoroisopropanol were obtained from Macklin Biochemical Co. (Shanghai, China). ZIF-8/PLA nanofibers was fabricated by electrospinning method using a blend of PLA and ZIF-8 (10 wt%). First, PLA was dissolved in hexafluoroisopropanol to a concentration of 10% (w/v) PLA solution via continuous magnetic stirring for 24 h. Subsequently, the ZIF-8 powder was homogeneously dispersed in deionized water by ultrasonication for 4 h in an ice-cold water bath. Different volumes of the ZIF-8 powder dispersion were added to the PLA solution and stirred in an ice-cold water bath to obtain a total concentration of 8% (wt./vol) ZIF-8/PLA solutions. The electrospun nanofibers were then generated. Briefly, the ZIF-8/PLA solution was filled in a 5-mL syringe with a 19-gauge needle. The syringe was placed vertically, and the distance between the tip of the syringe needle and collector was 20 cm. Electrospinning was performed for 10 h at 18 KV and a supply flow rate of 0.5 mL/h, the obtained membranes were then undergoing freeze drying.

### 2.2 Biomimetic apatite deposition on nanofibers

To accelerate the biomimetic apatite deposition, samples (PLA or ZIF-8/PLA nanofibers) were immersed in CaCl2 and K2HPO4 solutions. Then, nanofibers were immersed in 20 mL 0.2 M CaCl2 solution for 3 min and soaked in 30 mL ddH2O for 10 s, followed by soaking in 20 mL 0.2 M K2HPO4 solution for 3 min and in 30 mL ddH2O for another 10 s. The pretreatment assay was repeated three times. These alternately soaked samples were subsequently immersed in SBF for biomimetic apatite deposition (30 mL of SBF was poured into a 50 mL centrifuge tube containing six alternately soaked samples). The samples were kept at 37 °C for 14 days, and the SBF renewed every day to sustain a consistent ionic strength throughout the assay. The samples were removed from the SBF, gently washed with ddH_2_O, and subsequently lyophilized at −50 °C for 24 h under a vacuum. SBF was prepared according to the Kokubo protocol (pH 7.40, 37 °C) with final ionic concentrations (mM): Na^+^ 142.0, K^+^ 5.0, Mg^2+^ 1.5, Ca^2+^ 2.5, Cl^−^ 147.8, HCO_3_^−^ 4.2, HPO_4_^2–^1.0, SO_4_^2–^0.5. Variation in Ca^2+^ primarily modulates the nucleation rate rather than the eventual occurrence of apatite formation on ZIF-8 containing fibers.

### 2.3 Morphology and chemical composition of the nanofibers

The nanofibers were divided into four groups based on whether soaking in SBF or not: PLA nanofibers (PLA group), mineralized PLA nanofibers (mPLA group), ZIF-8/PLA nanofibers (ZIF-8/PLA group), and mineralized ZIF-8/PLA nanofibers (mZIF-8/PLA group).

All nanofibers were coated with platinum prior to observation. The surface morphology of the nanofibers was observed using a field emission scanning electron microscope (FESEM, Nova NanoSEM, Netherlands) at an accelerating voltage of 10 kV. The diameter of the nanofibers was calculated using representative images in OriginPro 9 software. The distributions of phosphorus, Calcium and Zinc in the nanofibers were recorded using an elemental mapping spectrometer.

### 2.4 Mechanical properties of the nanofibers

The mechanical properties of the nanofibers were determined using an all-electric dynamic test instrument (Instron, British) equipped with a load cell capacity of 100 N and tensile speed of 10 mm/min. The stress-strain curve was plotted by GraphPad Prism 8 Software, and Young’s modulus was calculated by OriginPro 9 Software.

### 2.5 Water contact angle, water uptake ability and water vapor transmission rates

The hydrophobicity of the nanofibers was measured using a contact angle goniometer (LSA100, LAUDA Scientific, Germany). The angle between the liquid droplet and solid surface was measured using a CCD video camera and a lens mounted on a viewing stage.

The water uptake ability of the nanofibers was determined as previous reported (Liu et al., 2023). nanofibers were tailored to 2 cm × 2 cm squares and weighed (W0). The samples were then immersed in deionized water at room temperature overnight. The surface water was carefully wiped away using filter paper, and the nanofibers were weighed again (W1). The water uptake ability of the nanofibers was calculated using Eq:

$$\mathrm{Water uptake ability}=\left(W1-W0\right)/W0\times 100\%$$

The moisture permeability of nanofibers was evaluated by measuring their water vapor transmission rates (WVTRs). Briefly, the nanofibers were cut into discs and mounted on the mouth of a cylindrical cup containing deionized water (exposed area A). All the groups were maintained in an incubator at 37 °C and 50% humidity for 24 h (the test period Δt = 1 day). The cup mass increase (Δm) was recorded. WVTR was calculated:

$$\text{WVTR}=\Delta m/\left(A\Delta t\right).$$

### 2.6 *In vitro* degradation and ions release assay

To quantitatively assess the biodegradability of the nanofibers, all the groups were weighed (W0) before incubation in PBS. At predetermined time points, the incubated membranes were removed, lyophilized, and reweighed (Wt). The degradation rate of the membranes was determined by assessing the percentage of the remaining mass and was calculated using Eq:

$$\mathrm{Remained mass}\left(\%\right)=\mathrm{Wt}/W0\times 100\%$$

The release of Calcium and zinc ions from nanofibers were performed by incubating them in 0.9% PBS at 37 °C. At predetermined time points, the solution was collected for analysis and replaced with an equal volume of a fresh solution. The concentrations of calcium and zinc ions were determined by inductively coupled plasma atomic emission spectroscopy (ICP-AES) (Prodigy Plus, Leeman, United States).

### 2.7 Biocompatibility of the nanofibers

Cytological experiments were performed using human dermal fibroblasts (HDFs; ATCC, United States). Cell culture was performed as previously described. The PLA, mineralized PLA, ZIF-8/PLA, and mineralized ZIF-8/PLA nanofibers were cut into 5 mm square shapes and sterilized with ethylene oxide at 37 °C overnight. The sterilized nanofibers were then placed at the bottom of 96-well culture plates and washed thrice with sterile PBS. Each well was seeded with 2 × 10^3^ cells.

The proliferation of cells on the nanofibers was assessed using a Cell Counting Kit-8 (CCK-8, Beyotime, China) after 72 h of incubation. Briefly, 10 μL of CCK-8 solution was added to each well containing 100 μL of complete DMEM and incubated at 37 °C for 2 hours. After incubation, 100 μL of the supernatant from each well was transferred to a new 96-well culture plate. The optical density (OD) of the solution was then measured at 450 nm using a microplate reader (BioTek ELx800, United States).

Cell viability on the nanofibers was measured using calcein-AM (CAM) and propidium iodide (PI) staining (Beyotime, China) at 24, 48 and 72 h of cell culture. The cytoplasms of live cells were stained green with CAM, while the cytoplasms of dead cells were stained red with PI. The fibroblasts were then observed under a confocal laser-scanning microscope (IX8 Olympus, Japan).

### 2.8 *In vivo* study on rats wound healing models

All procedures were carried out in compliance with the guidelines approved by the Institutional Animal Care and Use Committee (IACUC) of Westlake University (no. HZSY2024159-1). The wound healing model was conducted as previously described. Forty male Sprague-Dawley rats (age: 8 weeks; weight: 230–260 g) were housed separately in a standardized environment. All operations were performed under sterile conditions. Rats were anesthetized by inhalation of 2.5% isoflurane. The dorsal area was shaved, and a depilatory cream was used to completely remove the hair. The skin was disinfected, and full-thickness skin wounds (diameter: 10 mm) were made using a 10-mm biopsy punch on the dorsal skin.

The 40 wounds were randomly divided into four groups (n = 10): PLA, mineralized PLA, ZIF-8/PLA, and mineralized ZIF-8/PLA nanofibers. Incisions were covered with the respective nanofibers and then dressed with sterile transparent films (Tegaderm, 3M, United States). Wounds were wrapped using a self-adhering elastic bandage (Coban, 3M, United States), and images were recorded at 0, 3, 7, 10, and 14 days for further evaluation. The wound closing area was measured at these time points using Image-Pro Plus Software. At day 14 post-operation, each wound area was resected from the skin. The wound tissues were rinsed in PBS, fixed in 4% paraformaldehyde, and embedded in paraffin for further histological staining and analysis.

### 2.9 Histological analysis and immunohistochemistry staining

All mice were sacrificed 14 days post-operation. The tissues were fixed with 4% paraformaldehyde and stained with Hematoxylin-Eosin (H&E) to observe granulation and wound closure. Collagen deposition was assessed using Masson’s Trichrome staining and Sirius Red staining.

For immunobiological staining, paraffin-embedded tissue sections were incubated with rabbit anti-CD31 and anti-CD86 antibodies (Abcam, Cambridge, UK), followed by incubation with a horseradish peroxidase-conjugated secondary antibody (Dako, Glostrup, Denmark). To evaluate angiogenesis in the wound, CD31^+^ tubular structures were considered capillaries, and the capillary density in the healing wound area was quantified. To analyze the inflammatory response of the wound, CD86^+^ macrophages were counted.

### 2.10 Statistical analysis

Data were expressed as the mean value ± standard deviation from at least triplicate samples. Statistical analysis was performed using GraphPad Prism 8 Software. Differences between groups were analyzed using Student’s t-tests and one-way ANOVA. All data are represented as mean ± SD, and P value < 0.05 was considered to be statistically significant.

## 3 Results

### 3.1 Fabrication and characterization of the nanofibers

**FIGURE 1:**
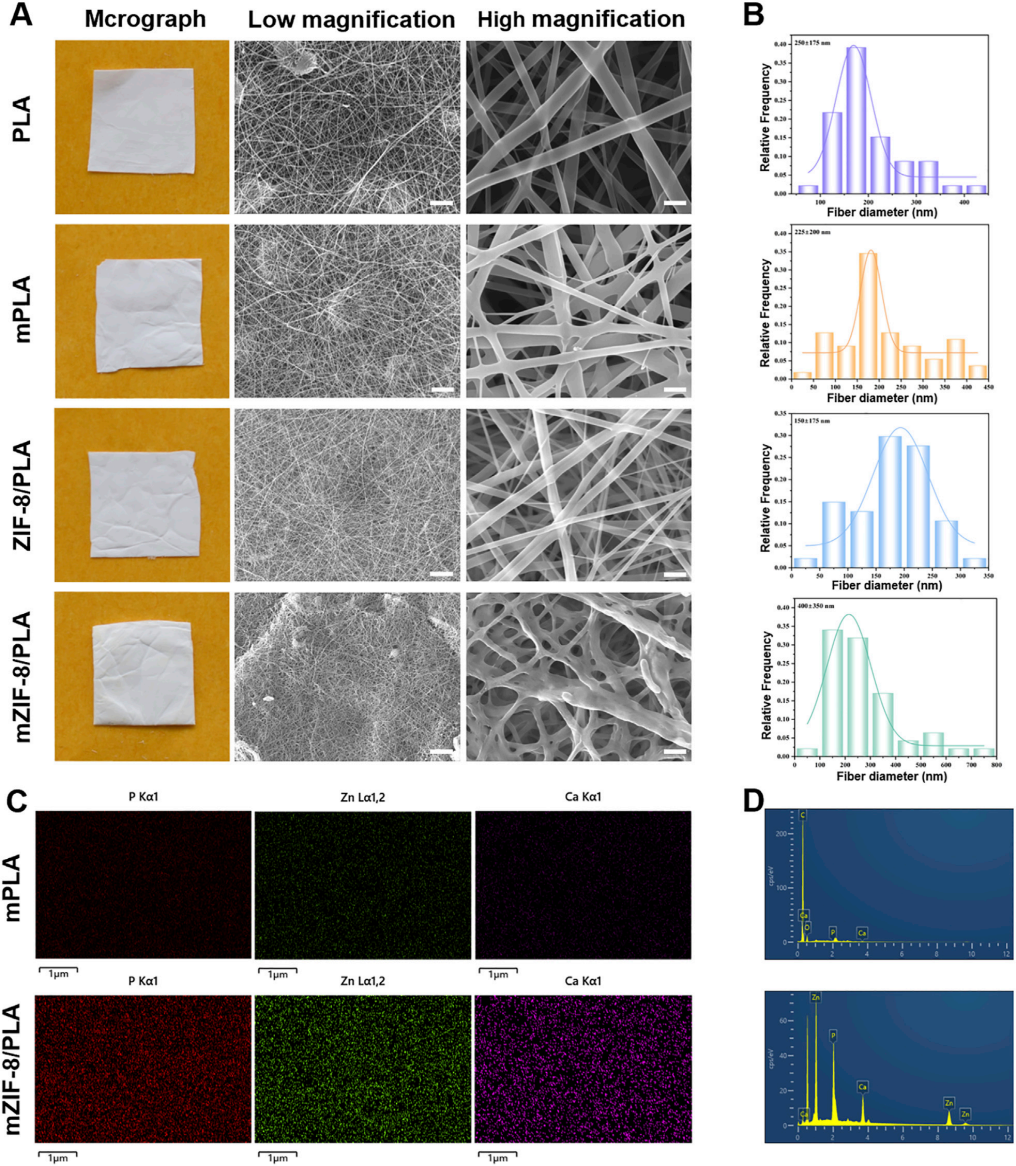
Characterization of morphology and elemental distribution in the nanofibers. **(A)** Macrographs and SEM images (scale bar: low magnification = 10 μm, high magnification = 5 μm); **(B)** Histograms of nanofibers’ diameter distribution; **(C)** Elemental mapping showed crystal deposition in mZIF-8/PLA nanofiber included Ca, Zn and P elements, whereas none of these elements were detected in mineralized PLA nanofiber; **(D)** Based on elemental mapping analysis of Ca, Zn and P elements peaks in mZIF-8/PLA.

The PLA, m-PLA, ZIF-8/PLA, and mZIF-8/PLA nanofibers showed white color (Figure 1A). Scanning electron micrographs of the nanofibers revealed that the addition of ZIF-8 had no effect on the formation of electrospun nanofibers. However, the nanofibers of mZIF-8/PLA were noticeably rougher at high magnification. The mZIF-8/PLA nanofibers were distributed randomly and uniformly to form a three-dimensional mesh structure that closely simulated the human extracellular matrix. It can also be observed that the interlaced pores covered most of the nanofibers, which is suitable for biomedical applications. Fresh TEM characterisation of the as-prepared ZIF-8 crystals confirmed well-defined rhombic-dodecahedral particles with a mean size of 50 ± 9 nm (Supplementary Figure S1), matching our earlier report and ensuring batch-to-batch consistency.

The diameter distribution of the nanofibers in each group is shown in Figure 1B. The mean diameters of the PLA, m-PLA, ZIF-8/PLA, and mZIF-8/PLA nanofibers were 250 ± 175 nm, 225 ± 200 nm, 150 ± 175 nm and 400± 350 nm, respectively. The addition of ZIF-8 did not affect the diameter of the nanofibers, whereas the induced mineralization in the SBF significantly increased the fiber diameter, especially in the mZIF-8/PLA group.

EDS mapping confirmed that the crystal deposits in the mZIF-8/PLA nanofibers were composed of calcium, phosphorus, and zinc, whereas no similar crystals were observed in the m-PLA nanofibers (Figures 1C,D).

### 3.2 Physiochemical properties of the nanofibers

#### 3.2.1 Mechanical strength of the nanofibers

**FIGURE 2:**
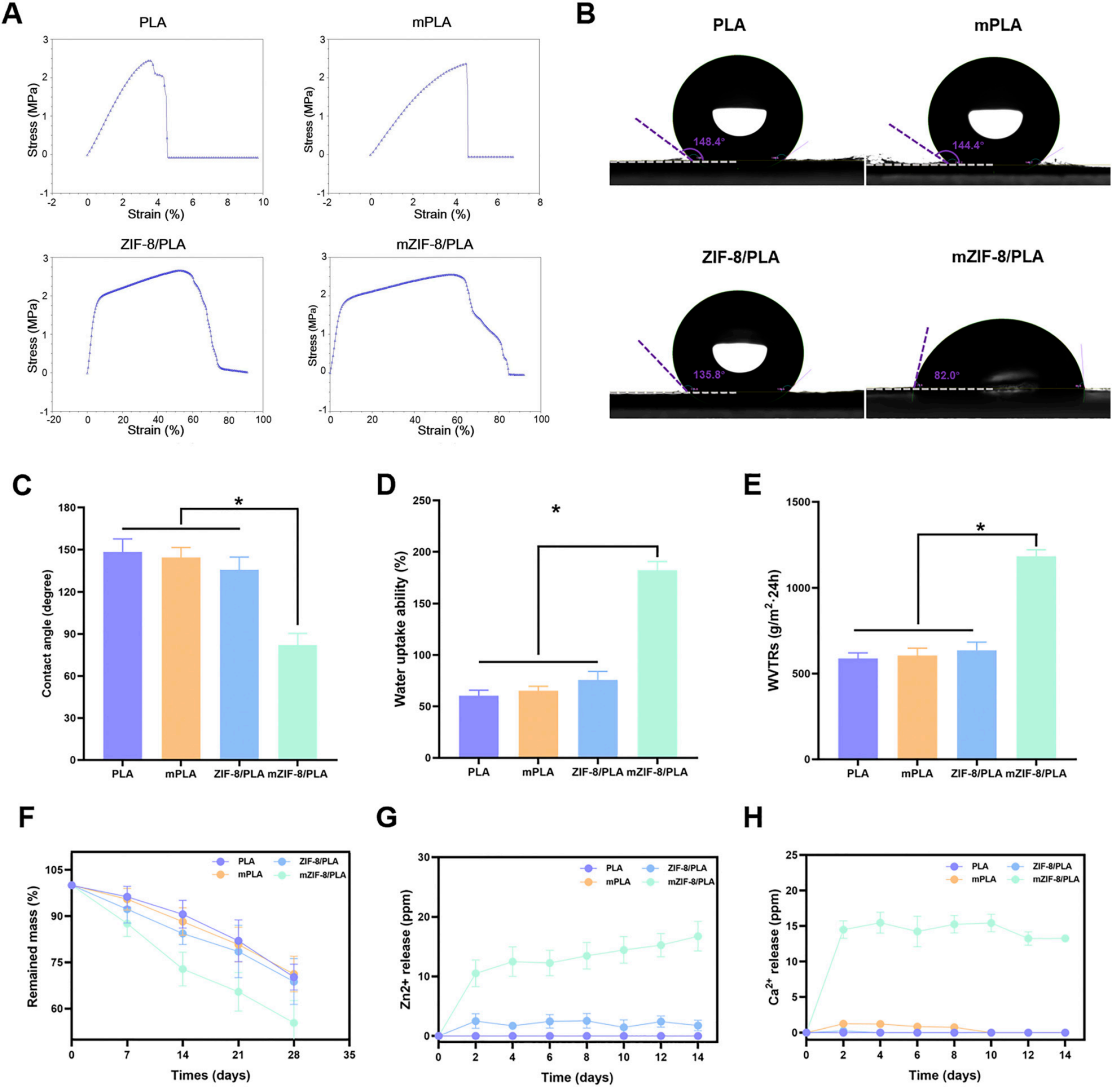
Mechanical strength, hydrophilicity, degradability and ions release of the nanofibers. **(A)** The stress-strain curves of the different nanofibers show that the tensile strains of the mZIF-8/PLA nanofibers were significantly higher than those of the other three groups, owing to the induced mineralization of ZIF-8 in the nanofibers; **(B)** Water contact angle tests demonstrated that the hydrophilicity of the mZIF-8/PLA nanofibers significantly increased; **(C)** Statistical graphics of water contact angles for each group; **(D)** Statistical graphics of water uptake ability (%); **(E)** Statistical graphics of water vapor transmission rates (WVTRs); **(F)** The degradability of the nanofibers, showing that the mZIF-8/PLA nanofibers experienced the fastest weight loss over time compared to the other three groups; **(G,H)** The release of Ca^2+^ and Zn^2+^ from the nanofibers over 14 days. (Data are shown as means ± SD; n = 3; *p < 0.05).

The mechanical strengths of the different nanofibers were investigated to determine whether the addition of ZIF-8 and induced mineralization affected the mechanical properties of the nanofibers. Figure 2A shows the stress-strain curves of the different nanofibers, which are linear within the 6% strain range. The tensile strains of the mZIF-8/PLA nanofibers were significantly higher than those of the other three groups, owing to the induced mineralization of ZIF-8 in the nanofibers.

#### 3.2.2 Hydrophilicity of the nanofibers

The water contact angle was measured to verify the hydrophobicity and hydrophilicity of the nanofibers. There was no significant difference in the water contact angle between the PLA and mineralized PLA groups (148.4 ± 1.46° and 144.4 ± 1.65°, respectively). The ZIF-8/PLA group had a water contact angle of 135.8 ± 1.75°, whereas the mineralized ZIF-8/PLA group had a significantly lower angle of 82.0 ± 3.45° (Figures 2B,C), indicating good hydrophilicity.

The water vapor transmission rates (WVTRs) and water uptake abilities of the different groups were further investigated. The mZIF-8/PLA nanofibers exhibited much greater WVTRs and water uptake abilities than the other three groups (Figures 2D,E). This finding suggests that the induced mineralization of ZIF-8 in the nanofibers enhances the hydrophilicity of the nanofibrous PLA membrane, making it more suitable for use as a wound dressing.

#### 3.2.3 The degradability and ions release of the nanofibers

The mZIF-8/PLA nanofibers experienced the fastest weight loss over time compared to other three groups and lost approximately 40% of their initial weight after 28 days *in vitro* (Figure 2F). Collectively, the WVTR (Figure 2E), high water uptake (Figure 2D) and controlled mass loss (Figure 2F) indicate that mZIF-8/PLA maintains a breathable yet sufficiently stable matrix for the healing window. The release of Ca^2+^ and Zn^2+^ mainly originates from the mZIF-8/PLA nanofibers (Figure 2G,H), In addition, almost no ions were released from the other three groups. The release of these two ions may partially explained why the mZIF-8/PLA nanofibers has biological activity.

### 3.3 *In vitro* biocompatibility and bioactivity of the nanofibers

**FIGURE 3:**
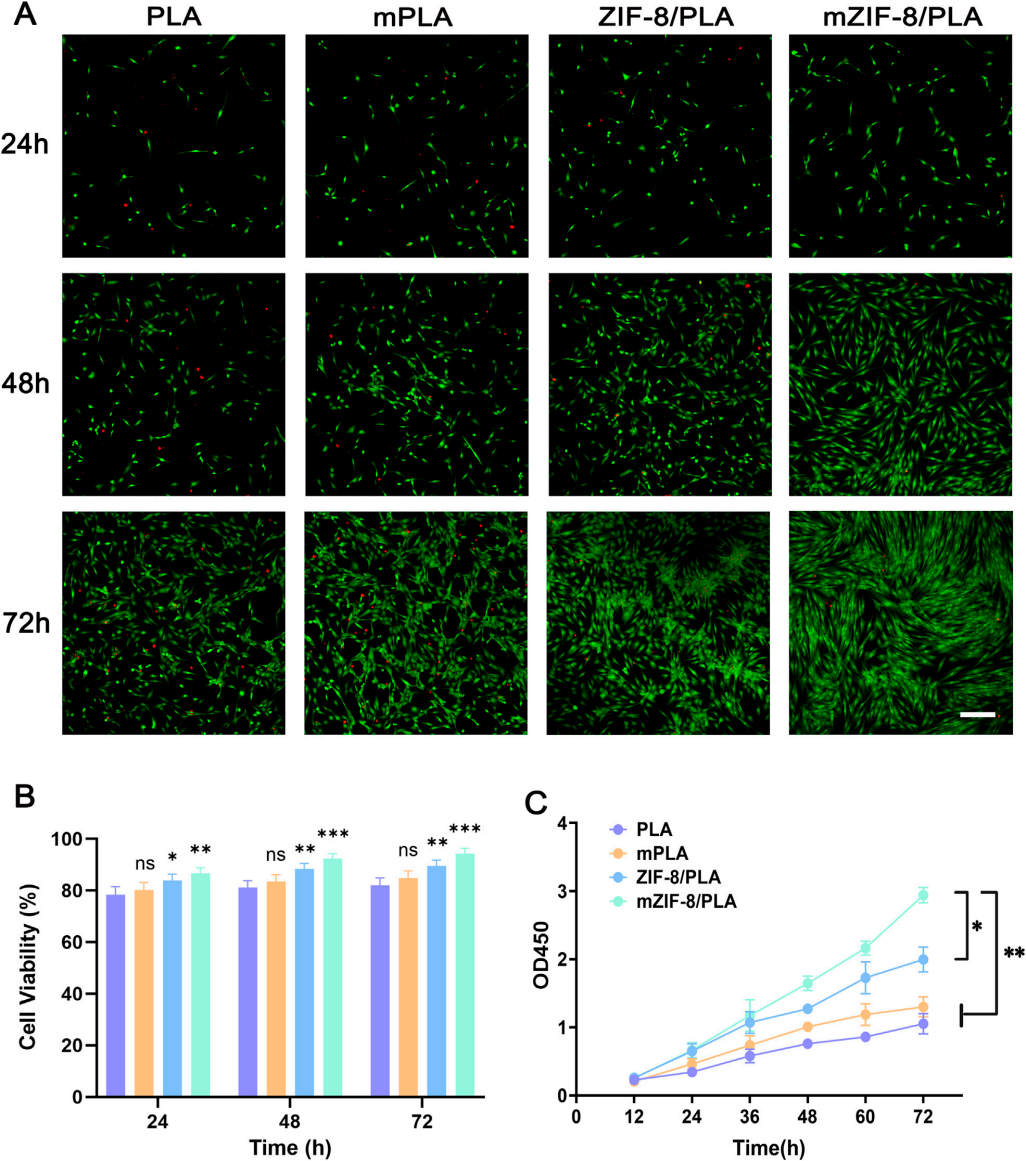
*In vitro* biocompatibility of the nanofibers. **(A)** Confocal fluorescent images for CAM/PI staining of human dermal fibroblasts on the nanofibers at 24 h, 48 h and 72 h of culture (scale bar = 200 μm); **(B)** Quantified cell viability (%) from image counts (n = 3); **(C)** CCK8 assay results of human dermal fibroblasts seeded on the nanofibers. The fibroblasts had the best cell proliferation rates on the mineralized ZIF-8/PLA nanofibers compared with other groups. (Data are shown as means ± SD; n = 3; *p < 0.05, **p < 0.01, ***p < 0.001, ns = not significant).

The biocompatibility of the nanofibers was evaluated using live/dead staining. Live fibroblasts were stained with calcein-AM (CAM) to produce green fluorescence, whereas dead fibroblasts were stained with propidium iodide (PI) to produce red fluorescence. The intensity of the CAM fluorescence on the mZIF-8/PLA nanofibers was significantly higher than that on the other three nanofibers after 48 h (Figure 3A). Compared with the PLA and mineralized PLA (m-PLA) nanofibers, there was a slight decrease in the PI fluorescence intensity in the ZIF-8/PLA and mZIF-8/PLA nanofibers after 72 h (Figure 3A). Quantified cell viability (%) showed consistently higher viability for mZIF-8/PLA at 24, 48 and 72 h (Figure 3B). Live/dead staining revealed notably better cell viability on the mZIF-8/PLA nanofibers, indicated by a greater proportion of live fibroblasts and a smaller proportion of dead cells. Similarly, fibroblasts cultured on mZIF-8/PLA nanofibers displayed accelerated proliferation, as assessed by the CCK-8 assay (Figure 3C). The mZIF-8/PLA nanofibers exhibited better cell proliferation rates compared to the PLA, m-PLA, and ZIF-8/PLA nanofibers. Overall, the mZIF-8/PLA nanofibers demonstrate good *in vitro* biocompatibility.

### 3.4 The mZIF-8/PLA nanofibers improved wound healing *in vivo*

**FIGURE 4:**
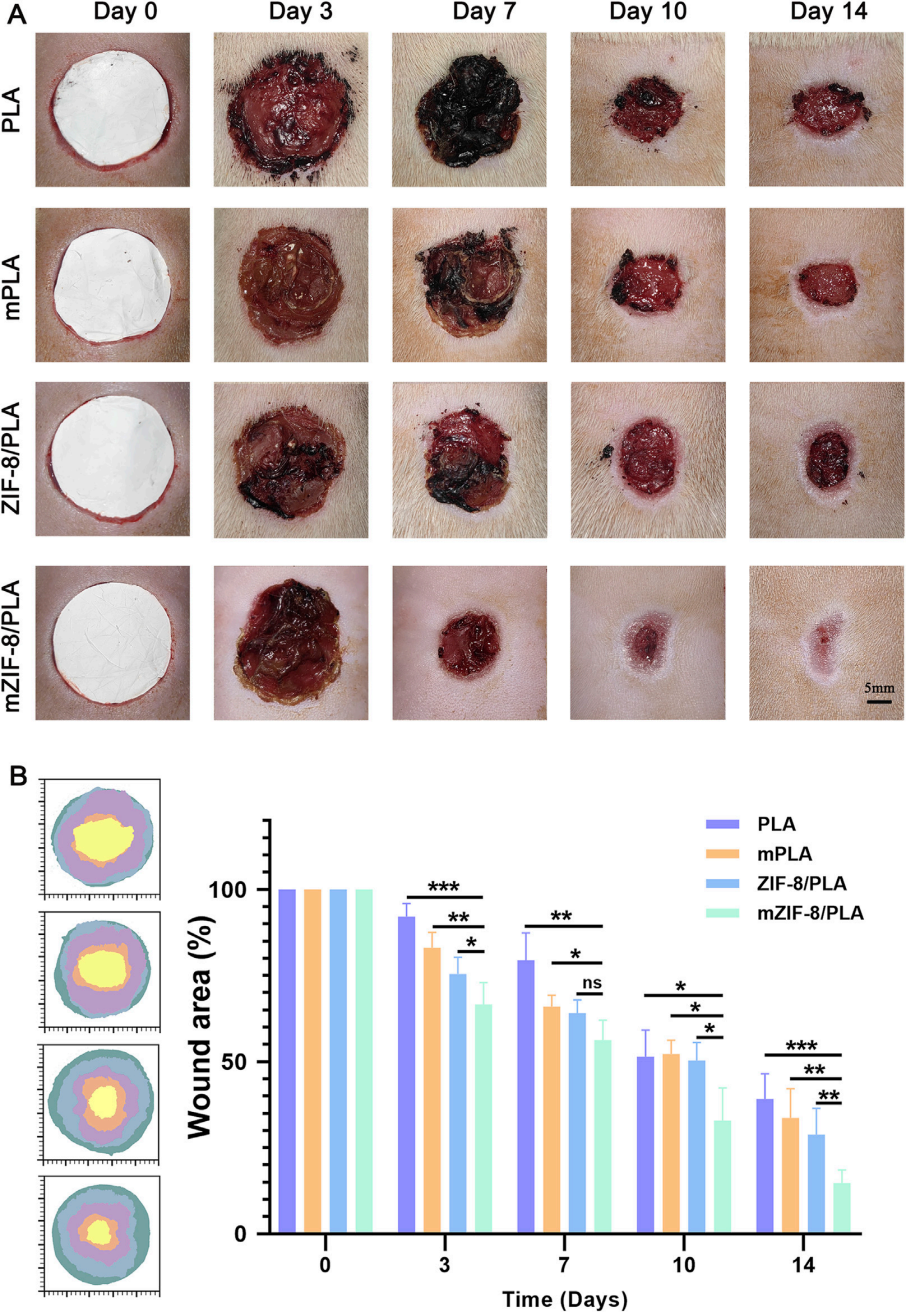
*In vivo* wound healing evaluation of the PLA, mPLA, ZIF-8/PLA, and mZIF-8/PLA nanofibers. **(A)** Photographic evaluation of wound repair in four groups on day 0, 3, 7, 10, and 14; **(B)** closure area (%) of the wound defect. The mZIF-8/PLA group achieved the best effect among four groups on day 3, 7, 10, and 14. (Data are shown as means ± SD; n = 6; *P < 0.05, **P < 0.01, ***P < 0.001).

The wound healing models were covered with PLA, m-PLA, ZIF-8/PLA, and mZIF-8/PLA nanofibers to evaluate the wound healing effect of the nanofibers *in vivo*. The wound healing process on days 0, 3, 7, 10, and 14 is shown in Figure 4A. The wound closure rates were calculated and are shown in Figure 4B. Before day 3, there was no significant difference in the percentage of wound closure areas among the four groups. However, by day 14, the percentage of wound closure in the mZIF-8/PLA group (86.62% ± 6.23%) was notably higher than that in the other groups. The PLA, m-PLA, and ZIF-8/PLA groups exhibited wound closure rates of 30.13% ± 9.55%, 53.2% ± 6.61%, and 61.32% ± 4.69%, respectively. Most of the wounds in the mZIF-8/PLA group showed significant closure by day 10, with re-epithelialization accomplished after 14 days. The mZIF-8/PLA group demonstrated the fastest wound closure rate among the four groups.

### 3.5 Histological analysis

**FIGURE 5:**
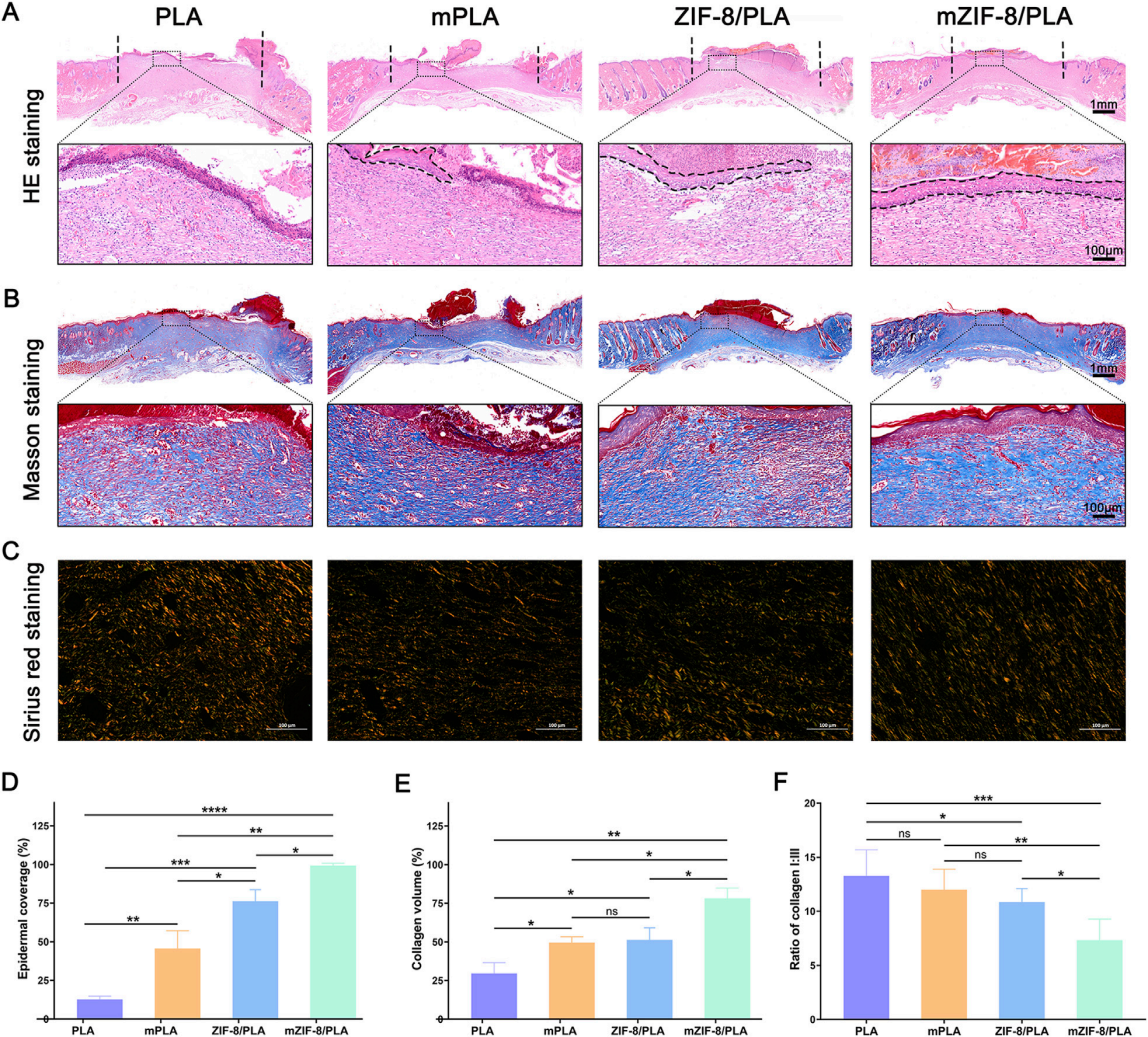
Histological analysis and collagen regeneration, maturation, and remodelling of wound tissues on 14 days. **(A)** H&E staining of tissue sections. The area between black dash lines: incomplete wound healing, and high magnified views of the central area are within the black solid lines; **(B)** Masson staining for representative wound beds on day 14; collagen deposition is stained blue; **(C)** Regenerated collagen content was analyzed using Picrosirius red staining under a polarized light microscope. Collagen type III is visualized in green color and collagen type I is visualized in orange/red color; **(D)** Statistic analysis of epidermal coverage in four groups; **(E)** The percentage of collagen volume to tissue volume was quantified according to the Masson staining results; **(F)** Ratio of collagen I:III in wound beds of each group. (Data are shown as means ± SD; n = 5; *P < 0.05, **P < 0.01, ***P < 0.001, ****P < 0.0001).

HE staining of wound areas covered with the four nanofibers is shown in Figure 5A. The area delimited by vertical lines, which lacks hair follicles and sebaceous glands, signifies regenerated granulation tissue. The healed wound will be covered by epithelium (dashed line); areas not covered by epithelium indicate that the wound has not healed. As shown in Figure 5A, the wound in the mZIF-8/PLA group is completely covered by epithelium, indicating complete wound healing. Statistical analysis of the length of the incomplete wounds demonstrated that the mZIF-8/PLA group had significantly enhanced wound healing (Figure 5D).

Collagen regeneration was examined by Masson’s trichrome staining (Figure 5B). On day 14, the mZIF-8/PLA group showed a significant increase in collagen deposition in the wound tissue compared to the other three groups (Figure 5E). The type of collagen fiber was investigated using Sirius red staining (Figure 5C), observed under a polarized light microscope. Most collagen fibers were identified as type I collagen, which appears red.

Additionally, wounds in the mZIF-8/PLA group presented a more regular and well-organized collagen fiber arrangement and a decreased ratio of type I/III collagen (Figure 5F), indicating that mZIF-8/PLA nanofibers reduced scar formation after wound healing.

### 3.6 Immunohistochemistry staining

**FIGURE 6:**
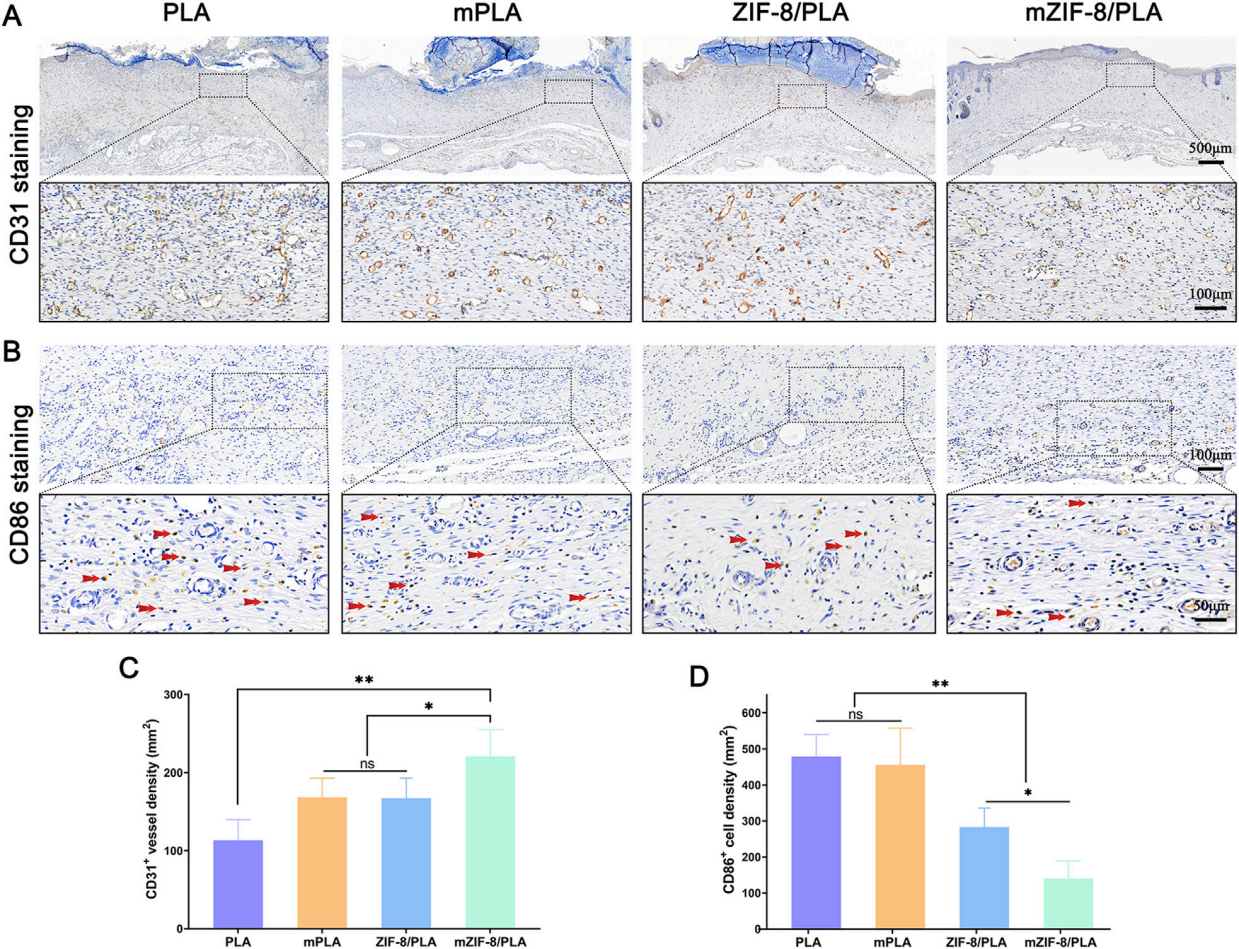
The effect of mZIF-8/PLA nanofibers on angiogenesis and inflammation in wound. **(A)** CD31-stained wound tissues on day 14 were shown via IHC assay; **(B)** CD86 staining results showed infiltration of macrophages/monocytes on day 14, and the red arrows indicate accumulation of CD86-positive cells in wound tissues; **(C)** Statistical analysis of number of CD31-positive vessels in the wound tissues; **(D)** The number of CD86-positive cells on day 14 was counted. (Data are shown as means ± SD; n = 5; *P < 0.05, **P < 0.01).

Immunohistochemical staining for CD31 was performed to investigate the effect of the different nanofibers on neovascularization at the wound site (Figure 6A). The number of blood vessels at the wound site in each group is shown in Figure 6C. Specifically, the wound area in the mZIF-8/PLA group had the highest number of blood vessels, whereas the PLA group had the least. The number of new blood vessels in the mZIF-8/PLA group was almost twice that of the other groups, suggesting that mineralized ZIF-8 notably enhanced neovascularization during the wound healing process. Immunohistochemical staining for CD86 was performed to investigate the effect of the different nanofibers on the inflammatory reaction at the wound site (Figure 6B). The density of CD86^+^ cells at the wound site for each group is shown in Figure 6D. The number of CD86^+^ cells in the mZIF-8/PLA group was significantly lower than those in the other groups, with no significant differences observed between the other three groups. This indicates that the mZIF-8/PLA nanofibers can significantly regulate the inflammatory response of wound tissue and prevent excessive inflammation.

## 4 Discussion

With the growing number of surgical procedures and chronic wounds, the promotion of skin regeneration in various wounds has become a major therapeutic challenge (Homaeigohar and Boccaccini, 2020). This study proposes PLA nanofibers loaded with ZIF-8, which induce biomimetic apatite deposition in simulated body fluids, this process results in the biomimetic mineralization of the surface of PLA nanofibers, enhancing their effectiveness for wound healing. *In vitro* and *in vivo* experiment revealed that the mineralized ZIF-8/PLA nanofibers enhances fibroblast proliferation, accelerates wound healing, promotes collagen regeneration, and relieves inflammation. Additionally, the type I/III collagen ratio in wound tissue was significantly downregulated, implying that scarless healing.

**FIGURE 7:**
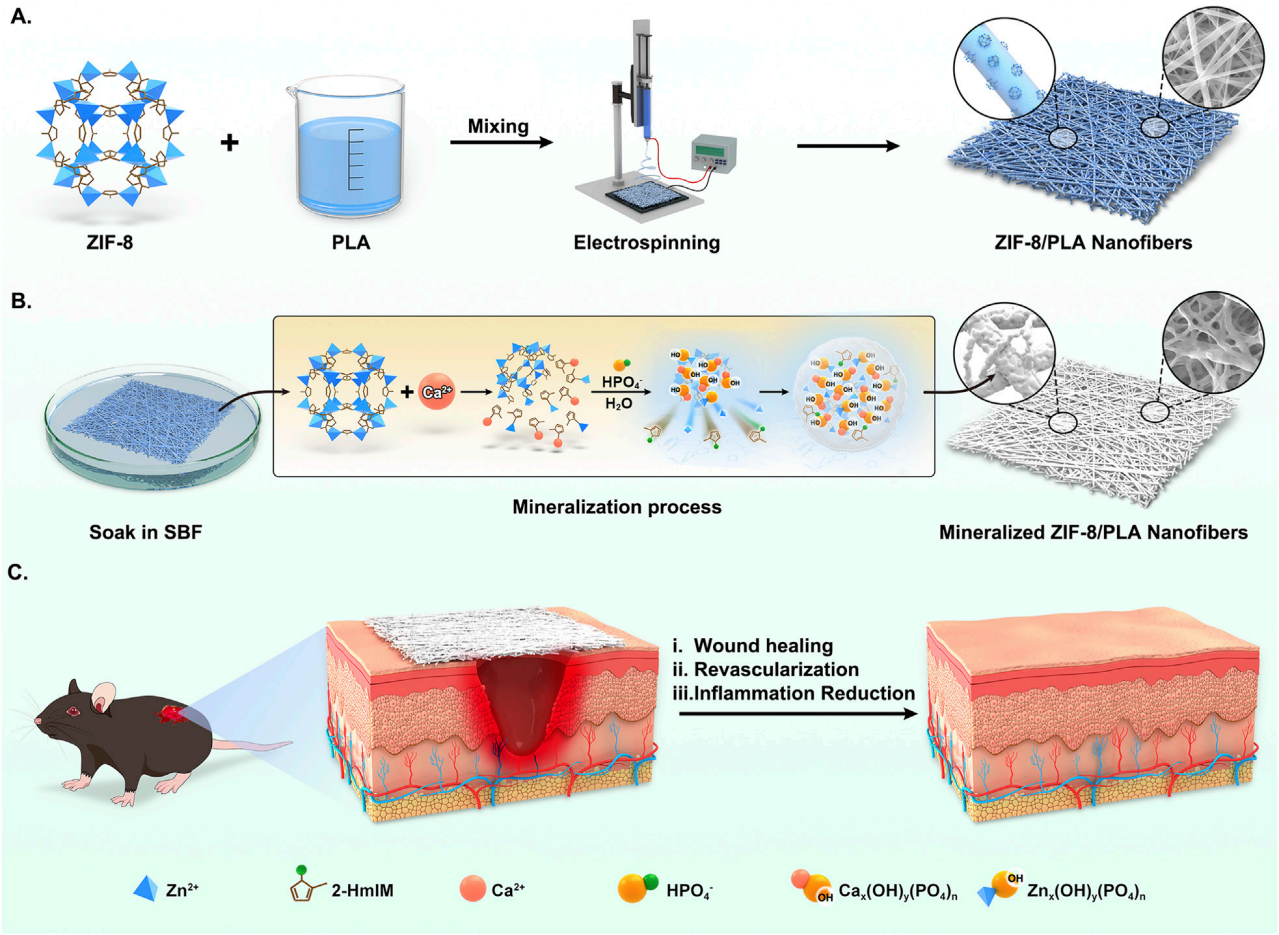
Schematic illustration of biological reinforced biomineralized ZIF-8/PLA nanofibers for wound healing. **(A)** The blend solution of ZIF-8 and PLA was first electrospun to fabricate the ZIF-8/PLA nanofibers. **(B)** The ZIF-8/PLA nanofibers was immersed in SBF solution to induce mineralization, resulting in the mineralized ZIF-8/PLA (mZIF-8/PLA) nanofibers. The mineralization process of ZIF-8 within the PLA nanofibers was illustrated: Calcium attack induced ZIF-8 degradation, providing Ca-P binding sites and causing apatite deposition. **(C)** The mZIF-8/PLA nanofibers accelerating wound healing of mouse dorsal skin by enhancing angiogenesis and reducing inflammation.

In previous research, we found that apatite deposition was formed after ZIF-8 degradation and induction in SBF, which could eventually generate mineralized pellets (Wang et al., 2023). Consistent with that study, the ZIF-8-induced biomineralization in our system follows a sequential process that is also experimentally supported by our current dataset (Figure 7). In practical terms, Zn-N bonds and hydrogen bonds in ZIF-8 slightly decompose due to Ca ion attack in the simulated body fluid (SBF) solution, leading to an early Zn^2+^ burst (Figures 2G–H) indicative of sacrificial framework dissolution. Consequently, an intermediate containing Zn and Ca ions bonded to a single 2-HmIM ligand is formed. Subsequently, HPO_4_^2−^ and H_2_O act as hydrogen donors to induce rapid protonation of 2-HmIM, resulting in the formation of excess calcium and zinc hydroxy phosphates. This protonation process may induce partial Zn ion release. Elemental mapping in this work (Figures 1C,D) confirms Ca and P deposition exclusively on ZIF-8-containing fibers, but not on PLA controls, supporting interfacial nucleation and growth of an apatite-like layer. Additionally, during the replacement process in SBF, the Ca content remained stably high, with the precipitation equilibrium always shifting towards the formation of Ca hydroxy phosphate. This layer growth is accompanied by increased fiber diameter, enhanced hydrophilicity (Figures 2B,C), and improved mechanical performance, altogether consistent with an emergent apatite coating. Due to electrostatic adsorption and covalent bonding, continuous agglomeration of mineralized pellets was observed. Therefore, our current physicochemical readouts (Zn^2+^ release, Ca/P co-localisation, wettability shift and mechanical strengthening) experimentally verify, in agreement with the proposed ZIF-8-induced biomineralisation mechanism.

PLA is derived from 100% renewable sources and is considered an eco-friendly material with good biocompatibility and controlled biodegradability. These properties make it a desirable candidate for wound dressings and tissue engineering applications (Avinc and Khoddami, 2010; Li et al., 2020). However, the poor biological activity of PLA limits its wide application. Therefore, researchers continue to develop composite formulations by incorporating PLA and other biologic agents to improve the biological activity of PLA-based materials while enhancing the ability to release bioactive molecules (Hajikhani et al., 2021; Khazaeli et al., 2020). To improve the poor biological activity while retaining the mechanical characteristics of PLA, ZIF-8 was incorporated into PLA. The ZIF-8/PLA nanofiber was synthesized through electrospinning in this research. The sample was then immersed in simulated body fluid (SBF) for a few days. As a result, obvious mineralized crystallization deposits were observed on the sample surface (Figure 1A). Based on EDS mapping images, P, Zn, and Ca ions showed a uniform distribution in the mineralized ZIF-8/PLA nanofibers. In contrast, these ions were almost not observed in the mPLA group, despite it also being soaked in SBF for several days (Figures 1C,D). This indicates that the mineralization on the fiber surface is induced by ZIF-8, not by the PLA itself or the SBF. Figure 2A depicts the stress–strain curve of the nanofibers, which evaluates their mechanical performance. Mineralisation not only significantly increases the breaking strength but also keeps the initial tensile modulus within the reported low-strain dermal range, indicating mechanical compatibility with native skin. The higher allowable strain before pronounced stiffening supports conformal contact and accommodation of routine periwound micro-extension, reducing interfacial shear. The strength enhancement is attributed to the greater average fibre diameter together with the apatite layer acting as a load-sharing, friction-enhancing sheath that dissipates stress without over-stiffening the scaffold.

Wettability, as an important physicochemical property of wound dressings, is a key prerequisite for absorbing wound exudate and maintaining an optimal level of moisture at the wound site (Bacakova et al., 2011). As shown in Figure 2B, PLA, being a hydrophobic material, had a water contact angle of approximately 148.4° for its nanofibers. When ZIF-8 was added, the contact angle decreased to 135.8°, but it was still not hydrophilic. After biomimetic mineralization, the water contact angle of the mZIF-8/PLA nanofibers was reduced to 82°, indicating hydrophilicity. Additionally, the mineralized ZIF-8/PLA membranes exhibited much greater water uptake ability (Figure 2D) and water vapor transmission rates (WVTRs) (Figure 2E) compared to the other three groups. Hydroxyapatite (HA) coating has received significant attention in the scientific community for the development of implants, it can be confirmed that HA can improve biocompatibility, enhance osteogenic activity, and HA as a polar molecule with hydrophilic properties could improve the hydrophilicity of materials (Cao et al., 2021; Qadir et al., 2021; Wang et al., 2019). Thus, we suspect that the hydroxyapatite formed on the surface of PLA nanofibers, induced by ZIF-8, contributes to the hydrophilicity and wettability of the material. This is advantageous for its application as a wound dressing.

Based on our results, the mZIF-8/PLA nanofibers exhibited superb cell activities and a lower apoptosis rate (Figure 3A). Anchoring-dependent cell division cannot be achieved without prior extension on the surface of the growth substrate, leading these cells to potentially undergo apoptosis (Kaniuk et al., 2020). The significantly increased roughness of the fiber surface, due to the deposition of apatite, promotes fibroblast attachment, growth, and differentiation in this research.

An ideal wound dressing should not only effectively accelerate the healing process, the quality of healing also deserve attention. The mZIF-8/PLA nanofibers exhibited an appropriate degradation profile over 28 days, making it advantageous for use as a wound dressing. Additionally, the slow release of bioactive substances such as calcium and zinc ions minimized potential toxicity to the body (Figures 2F,G). Based on current research, both of these ions play crucial roles in wound healing (Lin et al., 2017; Subramaniam et al., 2021). Zinc is an essential micronutrient in the human body, Zinc-dependent proteins play numerous indispensable roles within cells, such as transcriptional regulation (Zheng et al., 2015), DNA repair, apoptosis (Cho et al., 2016) and extracellular matrix (ECM) regulation (Tomlinson et al., 2008). Zinc has been shown effective for angiogenesis *in vivo* (Li and Chang, 2013) and angiogenesis played a crucial role in skin tissue regeneration, during wound repair angiogenesis performs the function of supplying essential nutrients and oxygen to the wound site, thus promoting granulation tissue formation (Liu et al., 2023; Wang X. et al., 2020), inhibited neovascularization is an important factor of refractory wound healing (Falanga, 2005). In the present study, we observed an increased capillary density in wounds treated with the mZIF-8/PLA nanofibers, as evidenced by histological evaluation of anti-CD31 immunohistochemical staining (Figures 6A,C). The continuous release of zinc ions from ZIF-8 degradation may have contributed to angiogenesis during wound healing. It has been found that zinc deficiency increases inflammatory cytokines and oxidative stress production in elderly subjects. Conversely, zinc supplementation can reduce plasma levels of oxidative stress markers and decrease the *ex vivo* production of inflammatory cytokines such as C-Reactive Protein (CRP) and IL-6 (Bao et al., 2010; Prasad, 2014). Dierichs et al. recently reported that zinc could participate in modulation of monocyte differentiation into pro-inflammatory (M1) or immune-regulatory (M2) macrophages, they discovered that zinc deficiency promote M1 phenotypes, while inhibiting M2 differentiation (Dierichs et al., 2018). A prolonged and heightened inflammatory response contributes to impaired healing, and regulating the inflammatory response can accelerate the healing process (Wood et al., 2014; Wu and Chen, 2016). This study showed that treatment of wounds with the mZIF-8/PLA nanofibers significantly reduces the infiltration of inflammatory macrophages (Figures 6B,D), this could contribute to accelerated wound closure. Apart from being a critical coagulation factor during hemostasis, the calcium ion has also been shown to act as a fundamental cue, directing the cellular functions of different types of cells during wound healing. Some studies have shown that calcium plays a vital role as the extracellular signaling molecule and intracellular second messenger in the morphology, proliferation, and collagen deposition of fibroblasts (Kawai et al., 2011). Navarro-Requena et al. conducted a study to examine the effects of extracellular calcium on skin fibroblasts cultured *in vitro*. They found that supplementation with extracellular calcium increased fibroblast metabolic activity, migration, MMP production, collagen synthesis, and cytokine release (Navarro-Requena et al., 2018). In this study, a significant increase in the synthesis and deposition of collagen was observed by Masson’s trichrome staining in wound tissues treated with the mZIF-8/PLA nanofibers (Figures 5B,E). These quantitative histological data, together with Sirius-red analysis (Figures 5C,F), indicate not only accelerated matrix accumulation but also a more regenerative ECM architecture. Similar collagen-promoting effects have been reported for mineralised ZIF-8/PLA membranes in bone-regeneration models (Wang et al., 2023). Besides the release of calcium ion, hydroxyapatite itself can also promote collagen regeneration, a phenomenon that has been confirmed in bone tissue engineering (Ramesh et al., 2018).

Our mineralized ZIF-8/PLA nanofibers exhibited excellent physicochemical properties, thereby promoting the wound healing process by enhancing cell proliferation, neovascularization, and collagen neogenesis. Additionally, the decreased inflammatory response and the reduced ratio of type I/III collagen in wound tissue indicate a potential for scarless healing.

## 5 Conclusion

In summary, this study demonstrates that mineralized ZIF-8/PLA nanofibers could promote wound healing. Moreover, ZIF-8 may serve as a bioactive additive that enables the surface modification of synthetic polymers, suggesting that it can be applied in *in-situ* skin regeneration.

## Data Availability

The original contributions presented in the study are included in the article/Supplementary Material, further inquiries can be directed to the corresponding authors.

## References

[B1] Avinc O. Khoddami A. J. F. C. (2010). Overview of Poly(lactic acid) (PLA) fibre. 42(1), 68–78.

[B2] Bacakova L. Filova E. Parizek M. Ruml T. Svorcik V. (2011). Modulation of cell adhesion, proliferation and differentiation on materials designed for body implants. Biotechnol. Adv. 29 (6), 739–767. 10.1016/j.biotechadv.2011.06.004 21821113

[B3] Bao B. Prasad A. S. Beck F. W. Fitzgerald J. T. Snell D. Bao G. W. (2010). Zinc decreases C-reactive protein, lipid peroxidation, and inflammatory cytokines in elderly subjects: a potential implication of zinc as an atheroprotective agent. Am. J. Clin. Nutr. 91 (6), 1634–1641. 10.3945/ajcn.2009.28836 20427734 PMC2869512

[B4] Bauzá A. , M. Tiddo J. Crystengcomm F. J. (2016). Towards design strategies for anion–π interactions in crystal engineering.

[B5] Bi H. Feng T. Li B. Han Y. (2020). *In vitro* and *in vivo* comparison study of electrospun PLA and PLA/PVA/SA fiber membranes for wound healing. Polym. (Basel) 12 (4), 839. 10.3390/polym12040839 32268612 PMC7240532

[B6] Bikle D. D. (2023). Role of vitamin D and calcium signaling in epidermal wound healing. J. Endocrinol. Invest 46 (2), 205–212. 10.1007/s40618-022-01893-5 35963983 PMC9859773

[B7] Bikle D. D. Xie Z. Tu C. L. (2012). Calcium regulation of keratinocyte differentiation. Expert Rev. Endocrinol. Metab. 7 (4), 461–472. 10.1586/eem.12.34 23144648 PMC3491811

[B8] Cao Z. Li L. Yang L. Yao L. Wang H. Yu X. (2021). Osteoinduction evaluation of fluorinated hydroxyapatite and tantalum composite coatings on magnesium alloys. Front. Chem. 9, 727356. 10.3389/fchem.2021.727356 34557474 PMC8453011

[B9] Castro-Aguirre E. Iñiguez-Franco F. Samsudin H. Fang X. Auras R. (2016). Poly(lactic acid)-Mass production, processing, industrial applications, and end of life. Adv. Drug Deliv. Rev. 107, 333–366. 10.1016/j.addr.2016.03.010 27046295

[B10] Chen H. L. Chung J. W. Y. Yan V. C. M. Wong T. K. S. (2023). Polylactic acid-based biomaterials in wound healing: a systematic review. Adv. Skin. Wound Care 36 (9), 1–8. 10.1097/asw.0000000000000011 37530559

[B11] Cheng H. Newton M. A. A. Rajib M. Zhang Q. Gao W. Lu Z. (2024). A ZIF-8-encapsulated interpenetrated hydrogel/nanofiber composite patch for chronic wound treatment. J. Mater Chem. B 12, 2042–2053. 10.1039/d3tb02683c 38315081

[B12] Cho J. G. Park S. Lim C. H. Kim H. S. Song S. Y. Roh T. Y. (2016). ZNF224, Krüppel like zinc finger protein, induces cell growth and apoptosis-resistance by down-regulation of p21 and p53 via miR-663a. Oncotarget 7 (21), 31177–31190. 10.18632/oncotarget.8870 27105517 PMC5058748

[B13] Dierichs L. Kloubert V. Rink L. (2018). Cellular zinc homeostasis modulates polarization of THP-1-derived macrophages. Eur. J. Nutr. 57 (6), 2161–2169. 10.1007/s00394-017-1491-2 28687933

[B14] Echeverría C. Muñoz-Bonilla A. Cuervo-Rodríguez R. López D. Fernández-García M. (2019). Antibacterial PLA fibers containing thiazolium groups as wound dressing materials. ACS Appl. Bio Mater 2 (11), 4714–4719. 10.1021/acsabm.9b00923 35021471

[B15] Falanga V. (2005). Wound healing and its impairment in the diabetic foot. Lancet 366 (9498), 1736–1743. 10.1016/s0140-6736(05)67700-8 16291068

[B16] Fan T. Daniels R. (2021). Preparation and characterization of electrospun polylactic acid (PLA) fiber loaded with Birch Bark Triterpene extract for wound dressing. AAPS PharmSciTech 22 (6), 205. 10.1208/s12249-021-02081-z 34286391 PMC8292269

[B17] Hajikhani M. Emam-Djomeh Z. Askari G. (2021). Fabrication and characterization of mucoadhesive bioplastic patch via coaxial polylactic acid (PLA) based electrospun nanofibers with antimicrobial and wound healing application. Int. J. Biol. Macromol. 172, 143–153. 10.1016/j.ijbiomac.2021.01.051 33450342

[B18] Hamed A. Ashraf S. Mostafa M. S. Khalaf M. Yousef H. Mourad I. (2023). Development of nanofibrous scaffolds containing polylactic acid modified with turmeric and hydroxyapatite/vivianite nanoparticles for wound dressing applications. Int. J. Biol. Macromol. 259 (Pt 1), 128624. 10.1016/j.ijbiomac.2023.128624 38061519

[B19] Han G. Ceilley R. (2017). Chronic wound healing: a review of current Management and treatments. Adv. Ther. 34 (3), 599–610. 10.1007/s12325-017-0478-y 28108895 PMC5350204

[B20] Homaeigohar S. Boccaccini A. R. (2020). Antibacterial biohybrid nanofibers for wound dressings. Acta Biomater. 107, 25–49. 10.1016/j.actbio.2020.02.022 32084600

[B21] Ignatova M. Manolova N. Markova N. Rashkov I. (2009). Electrospun non-woven nanofibrous hybrid mats based on chitosan and PLA for wound-dressing applications. Macromol. Biosci. 9 (1), 102–111. 10.1002/mabi.200800189 18855947

[B22] Jang E. J. Patel R. Patel M. (2023). Electrospinning nanofibers as a dressing to Treat diabetic wounds. Pharmaceutics 15 (4), 1144. 10.3390/pharmaceutics15041144 37111630 PMC10142830

[B23] Jayarama Reddy V. Radhakrishnan S. Ravichandran R. Mukherjee S. Balamurugan R. Sundarrajan S. (2013). Nanofibrous structured biomimetic strategies for skin tissue regeneration. Wound Repair Regen. 21 (1), 1–16. 10.1111/j.1524-475X.2012.00861.x 23126632

[B24] Kaniuk Ł. Krysiak Z. J. Metwally S. Stachewicz U. (2020). Osteoblasts and fibroblasts attachment to poly(3-hydroxybutyric acid-co-3-hydrovaleric acid) (PHBV) film and electrospun scaffolds. Mater Sci. Eng. C Mater Biol. Appl. 110, 110668. 10.1016/j.msec.2020.110668 32204096

[B25] Kawai K. Larson B. J. Ishise H. Carre A. L. Nishimoto S. Longaker M. (2011). Calcium-based nanoparticles accelerate skin wound healing. PLoS One 6 (11), e27106. 10.1371/journal.pone.0027106 22073267 PMC3206933

[B26] Khazaeli P. Alaei M. Khaksarihadad M. Ranjbar M. (2020). Preparation of PLA/chitosan nanoscaffolds containing cod liver oil and experimental diabetic wound healing in male rats study. J. Nanobiotechnology 18 (1), 176. 10.1186/s12951-020-00737-9 33256764 PMC7706058

[B27] Li H. Chang J. (2013). Bioactive silicate materials stimulate angiogenesis in fibroblast and endothelial cell co-culture system through paracrine effect. Acta Biomater. 9 (6), 6981–6991. 10.1016/j.actbio.2013.02.014 23416471

[B28] Li G. Zhao M. Xu F. Yang B. Li X. Meng X. (2020). Synthesis and biological application of polylactic acid. Molecules 25 (21), 5023. 10.3390/molecules25215023 33138232 PMC7662581

[B29] Liao M. Jian X. Zhao Y. Fu X. Wan M. Zheng W. (2023). Sandwich-like structure electrostatic spun micro/nanofiber polylactic acid-polyvinyl alcohol-polylactic acid film dressing with metformin hydrochloride and puerarin for enhanced diabetic wound healing. Int. J. Biol. Macromol. 253 (Pt 6), 127223. 10.1016/j.ijbiomac.2023.127223 37797847

[B30] Lin P. H. Sermersheim M. Li H. Lee P. H. U. Steinberg S. M. Ma J. (2017). Zinc in wound healing modulation. Nutrients 10 (1), 16. 10.3390/nu10010016 29295546 PMC5793244

[B31] Liu M. Wang X. Cui J. Wang H. Sun B. Zhang J. (2023). Electrospun flexible magnesium-doped silica bioactive glass nanofiber membranes with anti-inflammatory and pro-angiogenic effects for infected wounds. J. Mater Chem. B 11 (2), 359–376. 10.1039/d2tb02002e 36507933

[B32] Memic A. Abudula T. Mohammed H. S. Joshi Navare K. Colombani T. Bencherif S. A. (2019). Latest Progress in electrospun nanofibers for wound healing applications. ACS Appl. Bio Mater 2 (3), 952–969. 10.1021/acsabm.8b00637 35021385

[B33] Mutlu B. Çiftçi F. Üstündağ C. B. Çakır-Koç R. (2023). Lavandula stoechas extract incorporated polylactic acid nanofibrous mats as an antibacterial and cytocompatible wound dressing. Int. J. Biol. Macromol. 253 (Pt 3), 126932. 10.1016/j.ijbiomac.2023.126932 37729996

[B34] Navarro-Requena C. Pérez-Amodio S. Castaño O. Engel E. (2018). Wound healing-promoting effects stimulated by extracellular calcium and calcium-releasing nanoparticles on dermal fibroblasts. Nanotechnology 29 (39), 395102. 10.1088/1361-6528/aad01f 30039802

[B35] Parham S. Kharazi A. Z. Bakhsheshi-Rad H. R. Ghayour H. Ismail A. F. Nur H. (2020). Electrospun nano-fibers for biomedical and tissue engineering applications: a comprehensive review. Mater. (Basel) 13 (9), 2153. 10.3390/ma13092153 32384813 PMC7254207

[B36] Patel S. Srivastava S. Singh M. R. Singh D. (2019). Mechanistic insight into diabetic wounds: pathogenesis, molecular targets and treatment strategies to pace wound healing. Biomed. Pharmacother. 112, 108615. 10.1016/j.biopha.2019.108615 30784919

[B37] Prasad A. S. (2014). Zinc: an antioxidant and anti-inflammatory agent: role of zinc in degenerative disorders of aging. J. Trace Elem. Med. Biol. 28 (4), 364–371. 10.1016/j.jtemb.2014.07.019 25200490

[B38] Qadir M. Li Y. Biesiekierski A. Wen C. (2021). Surface characterization and biocompatibility of hydroxyapatite coating on anodized TiO(2) nanotubes via PVD magnetron sputtering. Langmuir 37 (16), 4984–4996. 10.1021/acs.langmuir.1c00411 33861930

[B39] Ramesh N. Moratti S. C. Dias G. J. (2018). Hydroxyapatite-polymer biocomposites for bone regeneration: a review of current trends. J. Biomed. Mater Res. B Appl. Biomater. 106 (5), 2046–2057. 10.1002/jbm.b.33950 28650094

[B40] Ribeiro N. Sousa A. Cunha-Reis C. Oliveira A. L. Granja P. L. Monteiro F. J. (2021). New prospects in skin regeneration and repair using nanophased hydroxyapatite embedded in collagen nanofibers. Nanomedicine 33, 102353. 10.1016/j.nano.2020.102353 33421622

[B41] Salimi E. (2021). Development of bioactive sodium alginate/sulfonated polyether ether ketone/hydroxyapatite nanocomposites: synthesis and *in-vitro* studies. Carbohydr. Polym. 267, 118236. 10.1016/j.carbpol.2021.118236 34119187

[B42] Schröder H. C. Tolba E. Diehl-Seifert B. Wang X. Müller W. E. (2017). Electrospinning of bioactive wound-healing nets. Prog. Mol. Subcell. Biol. 55, 259–290. 10.1007/978-3-319-51284-6_8 28238041

[B43] Subramaniam T. Fauzi M. B. Lokanathan Y. Law J. X. (2021). The role of calcium in wound healing. Int. J. Mol. Sci. 22 (12), 6486. 10.3390/ijms22126486 34204292 PMC8235376

[B44] Tajbakhsh S. Hajiali F. (2017). A comprehensive study on the fabrication and properties of biocomposites of poly(lactic acid)/ceramics for bone tissue engineering. Mater Sci. Eng. C Mater Biol. Appl. 70 (Pt 1), 897–912. 10.1016/j.msec.2016.09.008 27770967

[B45] Tan G. Wang L. Pan W. Chen K. (2022). Polysaccharide electrospun nanofibers for wound healing applications. Int. J. Nanomedicine 17, 3913–3931. 10.2147/ijn.S371900 36097445 PMC9464040

[B46] Tomlinson M. L. Garcia-Morales C. Abu-Elmagd M. Wheeler G. N. (2008). Three matrix metalloproteinases are required *in vivo* for macrophage migration during embryonic development. Mech. Dev. 125 (11-12), 1059–1070. 10.1016/j.mod.2008.07.005 18684398

[B47] Tran H. Q. Shahriar S. M. S. Yan Z. Xie J. (2023). Recent advances in functional wound dressings. Adv. Wound Care (New Rochelle) 12 (7), 399–427. 10.1089/wound.2022.0059 36301918 PMC10125407

[B48] Wang W. Cao N. Dong J. Boukherroub R. Liu W. Li Y. (2019). Chitosan/hydroxyapatite modified carbon/carbon composites: synthesis, characterization and *in vitro* biocompatibility evaluation. RSC Adv. 9 (40), 23362–23372. 10.1039/c8ra10396h 35514479 PMC9067253

[B49] Wang X. Deng M. Yu Z. Cai Y. Liu W. Zhou G. (2020a). Cell-free fat extract accelerates diabetic wound healing in db/db mice. Am. J. Transl. Res. 12 (8), 4216–4227. 32913499 PMC7476113

[B50] Wang Y. Yan J. Wen N. Xiong H. Cai S. He Q. (2020b). Metal-organic frameworks for stimuli-responsive drug delivery. Biomaterials 230, 119619. 10.1016/j.biomaterials.2019.119619 31757529

[B51] Wang W. Ding D. Zhou K. Zhang M. Zhang W. Yan F. (2021). Prussian blue and collagen loaded chitosan nanofibers with NIR-controlled NO release and photothermal activities for wound healing. J. Mater. Sci. and Technol. 34. 10.1016/J.JMST.2021.03.037

[B52] Wang B. Zeng Y. Liu S. Zhou M. Fang H. Wang Z. (2023). ZIF-8 induced hydroxyapatite-like crystals enabled superior osteogenic ability of MEW printing PCL scaffolds. J. Nanobiotechnology 21 (1), 264. 10.1186/s12951-023-02007-w 37563652 PMC10413775

[B53] Wood S. Jayaraman V. Huelsmann E. J. Bonish B. Burgad D. Sivaramakrishnan G. (2014). Pro-inflammatory chemokine CCL2 (MCP-1) promotes healing in diabetic wounds by restoring the macrophage response. PLoS One 9 (3), e91574. 10.1371/journal.pone.0091574 24618995 PMC3950222

[B54] Wu Y. S. Chen S. N. (2016). Extracted triterpenes from antrodia cinnamomea reduce the inflammation to promote the wound healing via the STZ inducing hyperglycemia-diabetes mice model. Front. Pharmacol. 7, 154. 10.3389/fphar.2016.00154 27378920 PMC4904009

[B55] Xia X. Song X. Li Y. Hou W. Lv H. Li F. (2022). Antibacterial and anti-inflammatory ZIF-8@Rutin nanocomposite as an efficient agent for accelerating infected wound healing. Front. Bioeng. Biotechnol. 10, 1026743. 10.3389/fbioe.2022.1026743 36277387 PMC9581157

[B56] Xiang T. Guo Q. Jia L. Yin T. Huang W. Zhang X. (2024). Multifunctional hydrogels for the healing of diabetic wounds. Adv. Healthc. Mater 13 (1), e2301885. 10.1002/adhm.202301885 37702116

[B57] Yin L. Tang Q. Ke Q. Zhang X. Su J. Zhong H. (2023). Sequential anti-infection and proangiogenesis of DMOG@ZIF-8/Gelatin-PCL electrospinning dressing for chronic wound healing. ACS Appl. Mater Interfaces 15 (42), 48903–48912. 10.1021/acsami.3c09584 37877332

[B58] Yu J. R. Navarro J. Coburn J. C. Mahadik B. Molnar J. Holmes J. H. t. (2019). Current and future perspectives on skin tissue engineering: key features of biomedical research, translational assessment, and clinical application. Adv. Healthc. Mater 8 (5), e1801471. 10.1002/adhm.201801471 30707508 PMC10290827

[B59] Zhang B. Chen J. Zhu Z. Zhang X. Wang J. (2023). Advances in immunomodulatory MOFs for biomedical applications. Small 20, e2307299. 10.1002/smll.202307299 37875731

[B60] Zheng J. Lang Y. Zhang Q. Cui D. Sun H. Jiang L. (2015). Structure of human MDM2 complexed with RPL11 reveals the molecular basis of p53 activation. Genes Dev. 29 (14), 1524–1534. 10.1101/gad.261792.115 26220995 PMC4526736

