# Supplementary material for: Accelerating wound healing by biomineralizing crystallization formed from ZIF-8/PLA nanofibers with enhanced revascularization and inflammation reduction
Source: Front Bioeng Biotechnol. 2025 Sep 5;13:1629244. doi: 10.3389/fbioe.2025.1629244 (PMC12447270; doi:10.3389/fbioe.2025.1629244)
Supplement: Supplementary file 1 [file DataSheet1.pdf]

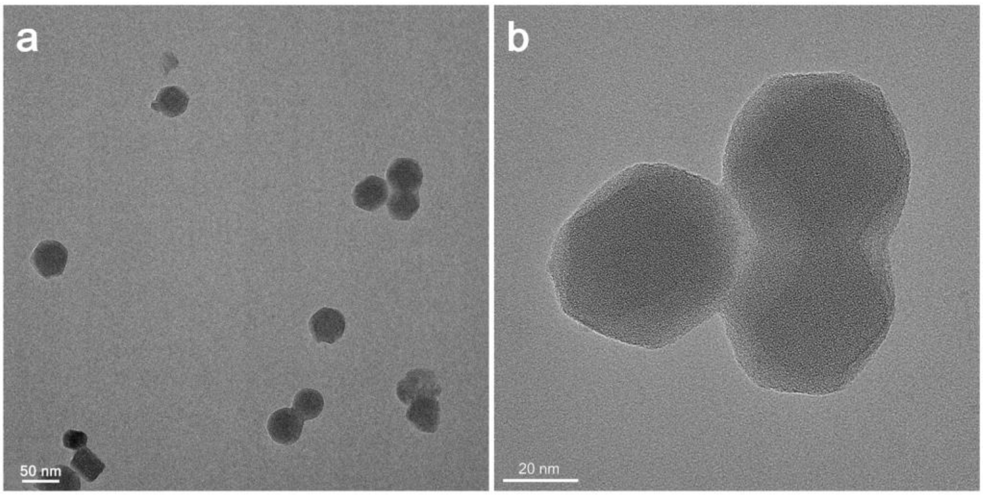

Figure S1 TEM analysis results of ZIF-8 particles: a. The particle size distribution of ZIF-8 is shown to be around average diameter of 50 ± 9 nm on low magnification (scale bar = 50nm). b. The particles display the expected rhombic-dodecahedral outline with smooth surfaces on high magnification (scale bar = 20nm).

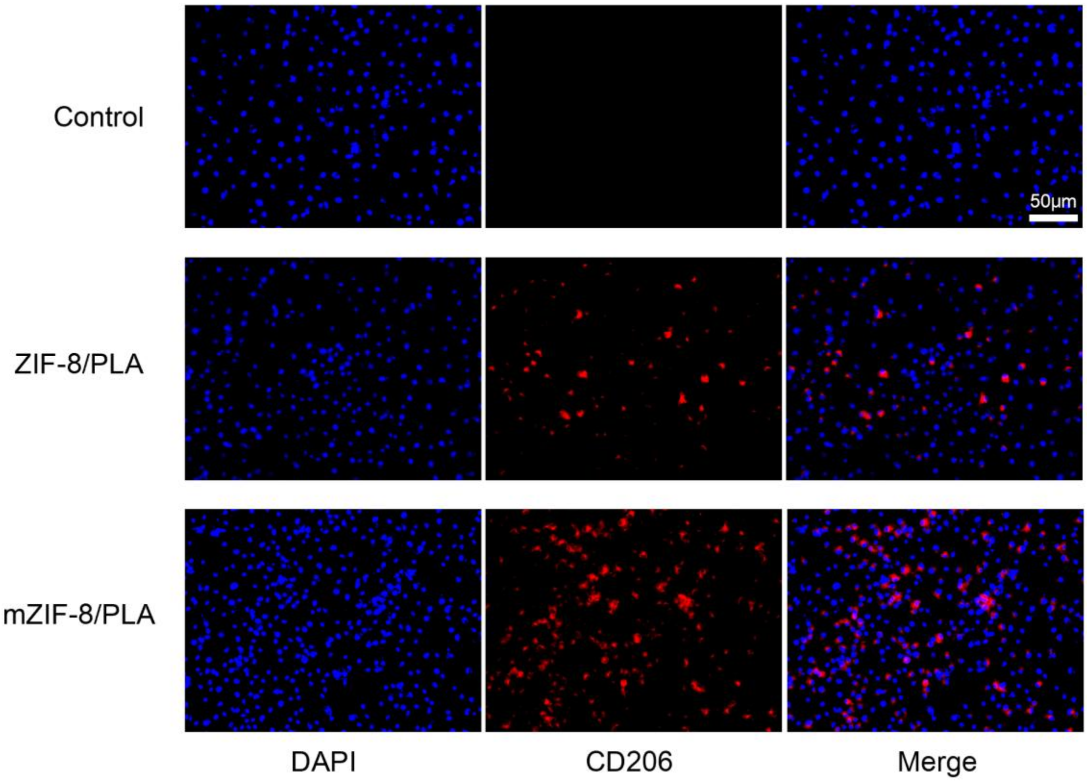

Figure S2 Immunofluorescent staining of CD206: Mineralized ZIF-8/PLA nanofibers promotes raw 264.7 cell differentiation to M2 macrophages in vitro.
